# Porcine Collagen Injection Therapy Affects Proximal Hamstring Tendinopathy in Athletes by Reducing Time to Return to Sport

**DOI:** 10.3390/sports13100359

**Published:** 2025-10-10

**Authors:** Matteo Baldassarri, Sarino Ricciardello, Diego Ghinelli, Luca Perazzo, Roberto Buda

**Affiliations:** 1 Clinic-Hospital “Villa Laura”, 40139 Bologna, Italy; sarinoricciardello@gmail.com (S.R.); lucaperazzo@hotmail.it (L.P.); 2 Clinic-Hospital “Villa Maria”, 47921 Rimini, Italy; dghinelli@omniway.sm; 3 Private Clinic-Hospital “Gruppioni”, 40036 Bologna, Italy; buda.ior@gmail.com

**Keywords:** porcine collagen injection therapy, hamstring injury, athletes, return to sport, VISA-H score, efficacy, safety

## Abstract

**Background:** Proximal hamstring tendinopathy (PHT) is a challenging overuse injury, particularly in athletes, characterized by deep buttock pain localized to the ischial tuberosity and often exacerbated by sports activities. This condition can impact an athlete’s performance, limiting high-level athletic activity. Return to sport (RTS) thus becomes a medical, physical, athletic, and economic necessity. Previous research has explored several conservative and injection-based therapies, but evidence regarding the efficacy of porcine collagen injections remains limited. Therefore, this study aims to compare the results obtained from ultrasound-guided porcine collagen injections versus a structured rehabilitation program in reducing time to return to sport (RTS) and improving Victorian Institute of Sport Assessment—Hamstring (VISA-H) scores with respect to athletes with clinically diagnosed PHT. Conservative approaches for PHT treatments include various options, such as physiotherapy, corticosteroids, plasma-rich-platelet, shockwave therapy, and collagen injection. Collagen demonstrated to be a validated option for tendinopathies treatment due its regenerative and restorative mechanism of action. **Methods:** Retrospective data were collected from twenty-eight athletes with a clinical diagnosis of PHT, confirmed based on pain provocation tests (Puranen–Orava, bent-knee, and modified bent-knee tests), who were divided into two groups: COL and REHAB. The VISA-H outcomes were recorded for all subjects. The COL group received three ultrasound-guided collagen injections at weekly intervals, plus standard care instructions. The REHAB group completed a progressive exercise program targeting hamstring and lumbopelvic stabilization. The primary outcomes were RTS time (days) and VISA-H scores at baseline and 8 weeks. Adverse effects were recorded. **Results:** The two groups of treatment were very homogeneous and showed parametric distribution concerning the biological and pathophysiological conditions. No adverse events were reported. The mean times to RTS were 57 and 72 days for COL and REHAB, respectively (*p* = 0.0083). The VISA-H results revealed better improvement for the COL group than the REHAB treatment (*p* < 0.0001), and the log-rank test showed a higher odds ratio (HR) for RTS, 5.35 (*p* = 0.0008), for the COL athletes. **Conclusions:** Ultrasound-guided porcine collagen injections, combined with standard care, significantly reduced RTS time and improved VISA-H scores compared with rehabilitation alone in athletes with PHT. However, a larger cohort of athletes might be needed to gather more information about this conservative treatment in PHT pathology.

## 1. Introduction

Proximal hamstring tendinopathy (PHT) is an overuse injury involving proximal insertion of the hamstring tendons at the ischial tuberosity [1,2]. It is characterized by deep buttock pain, sometimes radiating to the posterior thigh, exacerbated by activities such as sprinting, jumping, or prolonged sitting [1]. The main symptom of proximal hamstring tendinopathy (PHT) is lower gluteal pain, especially during running or prolonged sitting [3,4,5,6]. When athletes express acute pain in the posterior thigh, following a mechanical stimulus, often sudden (typical of hamstring strain injury; HSI), clinical examination is primarily focused on rehabilitation needs in order to return to sport (RTS), rather than on the search for the true diagnosis, resulting in problems of prognosis and athlete “post-traumatic shock syndrome” [7,8,9]. Unlike hamstring strain injuries (HSIs), which are acute muscle injuries, PHT involves chronic tendinopathy with degenerative changes. Therefore, the treatments should be focused specifically on PHT rather than HSI [1,2]. However, athletes who present pain in the posterior thigh require a differential diagnosis to confirm or exclude the presence of pathologies typical of this type of injury, which include proximal hamstring tendon avulsion, proximal hamstring tendinopathy, lumbar spine radiculopathy, and adductor muscle injury [7]. The causes triggering a PHT event are often controversial, and whether the traumatic event occurs following repeated and accumulated microscopic muscle damage or in response to a single (distinct) event that goes beyond the physical–mechanical limits of the biological muscle–tendon structure often remains unclear [10]. PHT can also result from constant wear of the tissue integrity associated with repeated damage over time, leaving the athlete vulnerable to triggers and therefore harmful sporting events. In other words, PHT can be caused by a single or macro-traumatic event (independent of the athlete’s muscular integrity, for example, hip flexion). Moreover, the mechanisms that lead to the formation of a PHT-type lesion probably involve a combination of several factors that include (1) a high load of high forces on the muscle–tendon unit, (2) excessive elongation compared to the structure of the muscle–tendon unit, and (3) the execution of high-velocity movements (e.g., sudden sprints) [6,11]. Several medical classification systems exist for muscle injuries. However, most of these classification methods are ten or more years old (O’Donoghue, Jackson, Takebayashi, Peetrons, Ryan, Durey and Rodineau, Stoller, Cohen, Chan, etc.) [12]; therefore, several expert groups have recently developed new classification systems:

- The Munich Consensus statement [13], which differentiates, among indirect muscle injuries, functional disorders (type 1a = fatigue-induced functional disorders; type 1b = delayed onset muscle soreness (DOMS); and types 2a and 2b = neuromuscular disorders of central or peripheral origin) and structural lesions (type 3a = minor partial muscle injury; type 3b = moderate partial injury; and type 4 = (sub) total muscle ruptures and tendon avulsions).
- The British Athletics Muscle Injury Classification (BAMIC) [14], which proposes to associate, on the one hand, the extent of the injury according to five progressive lesion stages based on magnetic resonance imaging (stage 0 = DOMS; stage 1 = minimal lesion in terms of longitudinal dimensions of the injury and percentage of fibers involved; stage 2 = moderate lesion; stage 3 = extensive lesion; and stage 4 = complete lesion) and, on the other hand, the site of the lesion (a = myofascial; b = muscular/musculotendinous; and c = intratendinous).
- The MLG-R classification, resulting from a collaboration between FC Barcelona and “Aspetar”, involves four aspects: M for mechanism of injury (direct, indirect “sprinting-type”, or indirect “stretching-type”); L for location (proximal, middle, or distal third of the muscle); G for grade (lesion stages 0 to 3); and R for number of recurrences (0 = first episode; 1 = first recurrence) [15].

A recent DELPHI consensus reported that specialists in the field used three classification systems for hamstring injuries, as follows: BAMIC (58%), the Munich Consensus (12%), and the Barcelona Consensus (6%) [16]. However, none are recommended for monitoring rehabilitation progress or assessing readiness to return to sport (RTS).

Diagnosis is clinical and based on characteristic history and physical examination findings, including pain provocation tests. The three most validated provocation tests are the Puranen–Orava test, the bent-knee stretch test, and the modified bent-knee stretch test. Positive findings are pain localized to the hamstring origin [17,18].

However, expert opinion concludes that more research is needed on the effectiveness of classification systems to prognosticate and guide treatment decision choice [16]. Traditional treatment methods for PHT are mostly comparable with those of other tendinopathies: they include rest and ice for symptom relief in the initial phase [19], reduction or pause of sports activity [20], non-steroidal anti-inflammatory drugs (NSAIDs) [21], physiotherapy, and continuous home exercise programs focusing on progressive eccentric hamstring strengthening and core stabilization [7,20,22,23]. Nonetheless, the surgical approach is also contemplated in PHT disorders [24,25,26]. However, surgical management is usually only performed when the conservative approaches fail, thus also prolonging RTS time by up to 12 months [25]. Therefore, conservative treatments are both recommended and useful when possible [27]. Novel therapeutic approaches have recently been recognized, including shockwave therapy (SWT) [28], ultrasound (US) therapy [29,30], ultrasound-guided corticosteroid injections [31,32], platelet-rich plasma (PRP) injections [33,34,35], and collagen injections [36,37]. US-guided corticosteroid injection of the tendons is used in PHT [38]; however, detrimental effects such as slowed long-term tissue healing caused by inhibition of collagen linkage, incomplete healing, and recurrent injury have been reported [39]. Additionally, symptoms often recur after initial good short-term results [10]. Furthermore, SWT [28], US therapy [29,30], and PRP injection demonstrated positive effects against the pathology spanning across 2–4 months of applications [33,34,35]. Tendinopathy is a chronic, painful tendon disease characterized by histological modifications such as disorganization of collagen (COL) [40], and the mechanical properties of tendons are based on the structure and composition of the extracellular matrix (ECM), which consists mainly of type I collagen [41]. The content of type I fibers inside the tendons plays a pivotal role in remodeling the ECM and in increasing the physical characteristic of tendons. Tenocytes support the mechanical adaptation of tendons by adapting to the mechanical stimuli imposed during loading, thus influencing the homeostasis of the extracellular matrix (ECM). Overall, type I collagen acts as a mechanical scaffold, constituting an effective therapeutic and regenerative tool to promote tendon healing in tendinopathies [42]. Randelli et al. demonstrated the effectiveness of porcine collagen in tenocytes in vitro, in which tenocytes cultured in a porcine collagen environment showed an increased proliferation rate and migration potential toward the site of lesion [41,43]. Additionally, recent evidence has demonstrated the positive effects of porcine collagen injections in the orthopedic field against greater trochanter pain syndrome (GTPS) [41,44]. Nevertheless, other studies revealed the efficacy of collagen injection therapy in musculoskeletal disorders in a short timeframe [37,45,46]. Other studies showed the positive effects of porcine collagen in reducing tendinopathies [47,48,49] and increasing body muscle strength [50]. Several positive effects have been documented, as noted above, with collagen applications representing a novel biological approach to treating tendinopathies and musculotendinous reconstructions [49]; they are also considered a cost-effective, easy-to-use, regenerative, and doping-free solution [42,43,51].

Indeed, time to RTS is the main goal of athletes and their medical staff because excessive prolongation of RTS affects athletes’ psychological behaviors, their performance, and the economic impact on sports teams [52].

The purpose of the present case series analyses is to compare the efficacy of the ultrasound-guided injection of porcine collagen treatment, in the sportive professionals affected by PHT, with respect to physiotherapy alone.

## 2. Materials and Methods

### 2.1. General Aspects and Eligibility Criteria

Data were acquired during the treatment timeframe. The data were acquired by the authors (S.R. and M.B.) to build an “ad hoc” database of clinical and anonymized personal data. An encrypted code was created at the beginning of this phase of the study, allowing for anonymity of the patients to be maintained. For data collection, the patients satisfied the following eligibility criteria:

- Clinical and MRI-confirmed diagnosis of proximal biceps femur tendinopathy:
  The diagnosis of PHT was based on patient history and clinical examination.
    MRI was used only when the diagnosis was uncertain, with confirmatory criteria including increased antero-posterior tendon thickness, increased peritendinous T2 signal, and bone marrow edema of the ischium.
- Age between 18 and 50 years.
- Competitive or semi-competitive sports activity.
- Symptom duration < 3 months (acute phase).
- Clinical diagnosis confirmed by at least two positive provocation tests (Puranen–Orava, bent-knee stretch, or modified bent-knee stretch).
- Localized pain at the ischial tuberosity on examination.
- Sportive or athletic individuals.
- No previous reconstructive or conservative surgical treatments.
- Had not benefited from infiltrative and/or other conservative treatment.
- No other concomitant pathologies within PHT.
- Completed treatment with MD-Muscle (Guna spa, Milano, Italy).

The exclusion criteria included the following:

- Complete tendon tears.
- Previous surgeries.
- Previous local injections or regenerative treatments.
- Rheumatologic or neurologic comorbidities.
- Known allergy to collagen or components of the product.
- Refusal to sign consent or poor compliance with follow-up.

### 2.2. Data Assessment

All data procedures were performed in agreement with the legal standards of human data manipulation per the Helsinki Declaration of 1975, updated in 2000 and 2008. The patient data employed for clinical evaluation concerned a total of 2 visits (T0 and T1). The T1 visit represents the final point for results collection. They were then compared with respect to the baseline data (T0). All patients included in the database first signed informed consent; after that, the treatments were performed.

### 2.3. Treatment and Technical Procedure

This case series used two groups of intervention:

(REHAB; Control group) The patients in this group received treatment through a rehabilitation program only, a structured, progressive exercise program supervised by a physiotherapist, focusing on isometric, concentric, and eccentric hamstring strengthening and lumbopelvic stabilization.

Rehabilitation program (REHAB group):

Phase 1 (weeks 0–2): isometric hamstring holds (5 × 45 s), lumbopelvic stabilization exercises (plank, side plank, bridges), avoiding positions causing tendon compression.

Phase 2 (weeks 3–6): progressive concentric and eccentric hamstring strengthening, including Nordic hamstring curls and Romanian deadlifts, performed at combined hip flexion (~110°) and knee flexion (45–90°).

Phase 3 (weeks 7–10): plyometric drills, sport-specific running drills, gradual return to training load.

Exercises included single-leg glute bridges, Romanian deadlifts, hip thrusts with 110° hip flexion, hamstrings with 45–90° knee flexion, progression from bodyweight to 70% 1RM, 3–4 sets of 8–12 repetitions, controlled speed, and the introduction of plyometric exercises in the final phase. Progression was individualized according to pain response, aiming for pain-free strength testing and negative provocation tests before return to sport.

(COL; Collagen) The patients in this group received 3 weekly ultrasound-guided injections of 4 mL each (MD-Muscle, porcine collagen, GUNA) in addition to an individualized rehabilitation program, as is mentioned above. All COL patients received the collagen injection in their proximal biceps femur tendon through ultrasound-guided injection with a 22G needle (23mm in length). Before the injection, the target area was disinfected with alcohol or another antiseptic solution. The treatment consisted of 1 administration of porcine collagen (MD-Muscle 4 mL; 2 vials)

**Figure 1:**
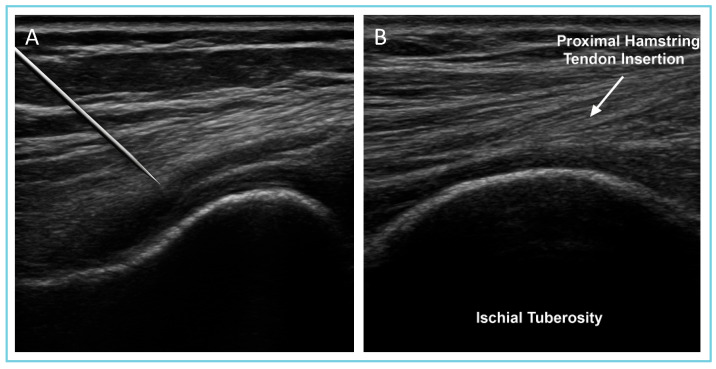
Ultrasound-guided injection of MD-Muscle (porcine collagen) in the proximal hamstring region. (**A**) The physician uses an ultrasound probe for real-time needle guidance to ensure precise delivery into the proximal hamstring tendon. (**B**) Magnification of image (**A**) showing the PHT region (arrow) and ischial tuberosity (bottom of the figure). The labeled structures highlight the muscle tissue and the hamstring tendon. This imaging confirms the correct positioning of the needle under ultrasound guidance.

The procedures were performed by a specialist in musculoskeletal pathology through an ultrasound machine. The correct deposition was confirmed via ultrasound and MRI imaging (Figure 1).

Concomitant care: No other injection therapies, shockwave therapy, or NSAID use was permitted during the study period. Both groups were allowed to continue low-load aerobic activities (cycling, swimming) if pain-free. These clarifications ensure transparency and confirm that both groups received an evidence-based rehabilitation program, with the only difference being the addition of porcine collagen injection in the COL group.

### 2.4. Follow-Up and Score Evaluation

Follow-up took place until the athlete was deemed to RTS. In any case, follow-up duration was established for a period of no less than 56 days, i.e., 8 weeks. The assessment of the clinical status of the athletes was assessed through the Victorian Institute of Sport Assessment (VISA questionnaire)—Hamstring (-H) questionnaire, which is a self-report clinical outcome measure used in patients with proximal hamstring tendinopathy (PHT) [18]. It consists of eight questions that measure the domains of pain, function in daily life, and sporting activities. This assessment was performed at baseline before the start of treatment (T0) and at least 8 weeks after treatment. On a weekly basis, the athletes’ clinical conditions were monitored by evaluating the following biophysical parameters as well: pain, functionality, and general condition for return to sport (RTS) [5,29,53].

### 2.5. Statistical Procedures

#### 2.5.1. General Methodology

All data were listed and sorted by gender, analysis population, and type of sport. All summary tables of the efficacy data were structured with a column for each target and were annotated with the total population size relevant to that table/treatment, including any missing observations. The continuous variables were summarized using the following descriptive statistics: *n* (number), mean, standard deviation, median, maximum, and minimum. The frequency and percentages (based on the non-missing sample size) were reported for all categorical measures. The data generated in this study were recorded in a study-specific electronic system, and the original rows of data can be made available on demand. After the completion of data entry in the system and the resolution and closure of all discrepancies, the database was blocked to avoid any further modifications. After quality checks, the SAS format database was used for statistical analysis.

#### 2.5.2. Study Variables

For participants’ demographic and clinical data documentation, the following variables were documented: year of birth, age (years), gender (male/female), weight, height, BMI calculation, pain evaluation (VISA-H score), and return to sport (RTS) time.

#### 2.5.3. Analytical Test Application (ATA)

GraphPad 8.0 version for Apple Computer was used for statistical analysis (PRISM, San Diego, CA, USA). The Shapiro–Wilk test was performed to determine whether the data were parametrically distributed. Both W- and *p*-values were calculated for all data distributions. Student’s Test (parametric and paired) was used to compare treatment results at the T0 and T1 visits. These analyses were performed to evaluate VISA-H output in both the COL and REHAB treatments. These tests were used to determine (if any) the differences between before and after treatment in the degree of clinical improvements in PHT, while Student’s Test (parametric and unpaired) was used to compare the values of VISA-H at T0 in both groups of treatment (COL and REHAB) and time to return to sport (RTS). All the parameters measured in this study were evaluated using the classical descriptive statistics: mean, SD, minimum and maximum, and frequencies (for qualitative variables). The log-rank test was used to assess the time-to-event outcomes (hazard ratio; (95% CI). For quantitative analyses, all statistical results were considered significant if the *p*-value was less than 0.05 (*p* < 0.05).

## 3. Results

### 3.1. Athlete Distribution

**Figure 2:**
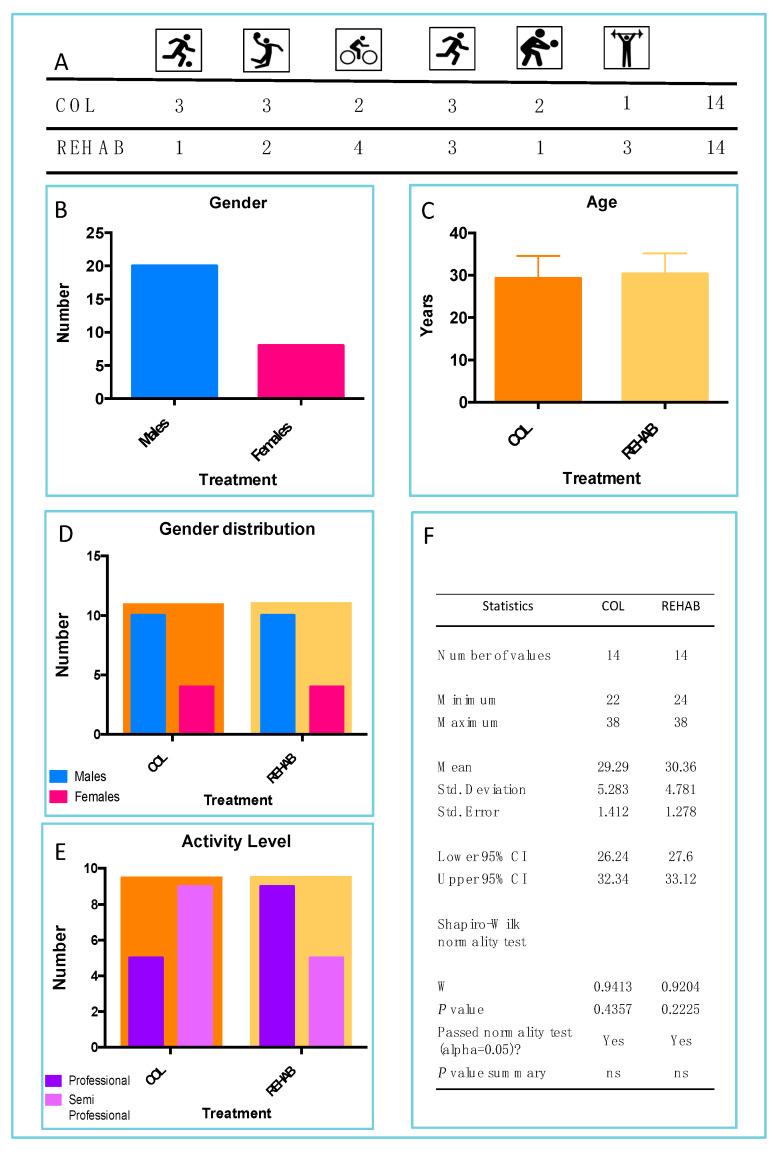
Data representation concerning athletes distribution. (**A**) Sport activities; from left to right, Football, Basketball, Cycling, Triathlon, Volleyball, and Crossfit. (**B**) Mean age concerning the treatment: COL (orange) and REHAB (peach). Gender distribution concerning (**C**) global distribution; (**D**) type of treatment and (**E**) level of sport activity. (**F**) Statistics concerning the parametric distribution of athletes (age).

In total, 28 athletes were included in this study (Figure 2), 2 groups of 14 athletes each: the COL and REHAB treatments. The athletes belong to six different sports disciplines: Football, Basketball, Cycling, Triathlon, Volleyball, and Crossfit (Figure 2A). In total, 20 were male and 8 were female (Figure 2B). The two treatment groups demonstrated parametric data distribution for age (mean age 29.29 vs. 30.36; Figure 2C) and identical distribution of data concerning global gender distribution for treatment (Figure 2D) and gender distribution for level of sport activities (Figure 2E). Indeed, the two groups of analyses (COL and REHAB) were two homogeneous groups. The results of the Shapiro–Wilk test for parametric data distribution are reported in Figure 2F.

### 3.2. VISA-H Questionnaire Scores at T0 Visit

**Figure 3:**
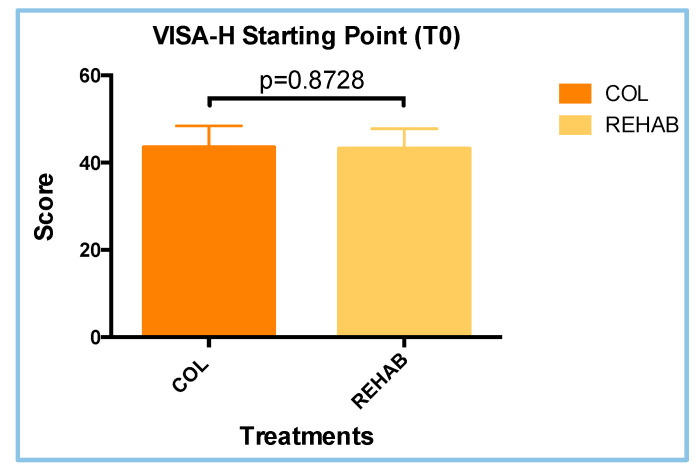
Results concerning the VISA-H scores at the starting point (T0). The differences are not statistically significant (*p* = 0.8728; *T*-test analyses; parametric; unpaired).

In order to compare the statistical differences between the two treatment groups, the VISA-H questionnaire scores were evaluated for all athletes before the start of the treatment, at the T0 visit. The questionnaire scores for the COL and REHAB groups were 43.50 ± 1.317 and 43.21 ± 1.219, respectively (Figure 3). These differences did not show a significant value through the parametric unpaired *T*-Test (*p* = 0.8728). For this reason, the groups were considered identical at T0. In addition, the Shapiro–Wilk test revealed that the two treatment groups had parametric distributions (Table 1).

### 3.3. Follow-Up

The duration of follow-up (FU) with the athletes ranged from 7 to 14 weeks. The maximum FU in the COL treatment was 65 days, while the maximum FU in the REHAB protocol was 95 days.

### 3.4. Return to Sport (RTS) Results

**Figure 4:**
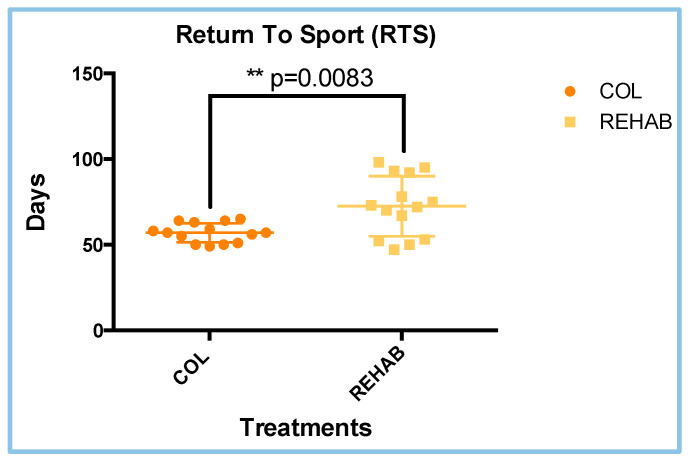
Return to sport (RTS) results. The differences between COL and REHAB were statistically different (** *p* = 0.0083; *T*-Test analyses; parametric; unpaired).

Time to return to sport (RTS) is the most important parameter from both the clinicians’ and athletes’ points of view. The RTS analyses revealed that the COL and REHAB treatments had different time intervals to RTS after treatment. The COL group had a mean time to RTS of 57 ± 5.588 days (about 8 weeks), while the REHAB group had a mean of 72.50 ± 17.51 days. The minimum and the maximum values for time to RTS were 47 and 95 days, respectively (concerning both treatments). The confidence intervals (CI; 95%) were 53.77–60.23 days for the COL treatment and 62.30–82.61 days for the REHAB treatment. These analyses revealed statistical differences between the two treatments (*p* = 0.0083) based on parametric and unpaired *T*-Tests (Figure 4). Analyses of the parametric distribution are reported in Table 2. These data suggest that the COL treatment improves the time to RTS, in all sports, over the REHAB one.

### 3.5. VISA-H Score Results in the COL Treatment

**Figure 5:**
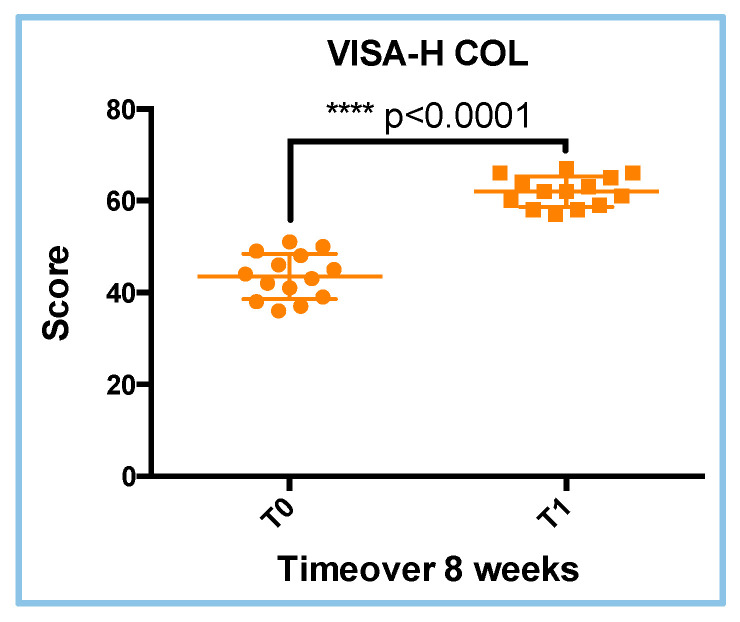
VISA-H scores in the COL treatment. The differences between the T0 and T1 visits were statistically different (**** *p* < 0.0001; *T*-Test analyses; parametric; paired).

The VISA-H questionnaire scores were used to evaluate the clinical condition of all athletes in the COL treatment during follow-up until the time to RTS. Comparisons between the starting point (T0) and the end point (T1) were made separately in both groups of treatment (COL and REHAB). The mean scores in the COL group were 43.50 ± 4.926 and 62.00 ± 3.305 at the T0 and T1 visits, respectively. The VISA-H scores showed statistical differences through the *T*-Test (parametric and paired test) analyses (*p* = 0.0001; Figure 5). Analyses of the parametric distribution of data are reported in Table 3. These analyses suggest that the COL treatment improves the VISA-H scores at the T1 visit (Figure 5).

### 3.6. VISA-H Score Results in the REHAB Treatment

**Figure 6:**
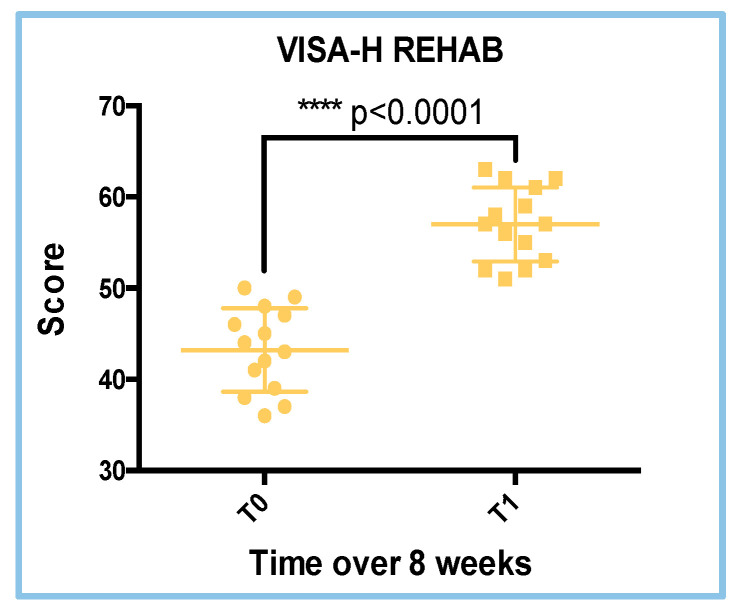
VISA-H scores in the REHAB treatment. The differences between the T0 and T1 visits were statistically different (**** *p* < 0.0001; *T*-Test analyses; parametric; paired).

The VISA-H questionnaire scores were used to evaluate the clinical condition of all athletes in the REHAB treatment during follow-up until the time to RTS. The mean scores in the REHAB group were 43.21 ± 4.560 and 67.00 ± 4.057 at the T0 and T1 visits, respectively. The VISA-H scores showed statistical differences through *T*-Test (parametric and paired test) analyses (*p* = 0.0001; Figure 6). Analyses of the parametric distribution of data are reported in Table 4. These data suggest that the COL treatment does not improve VISA-H scores at the T1 visit, as was found for the COL treatment (Figure 6).

### 3.7. VISA-H Analyses (Delta and Speed Evaluation)

**Figure 7:**
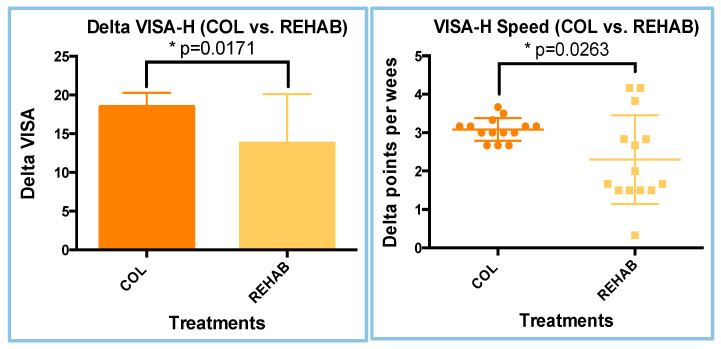
Results of the delta VISA-H (**left**) and VISA-H speed (**right**) analyses.

To further evaluate whether the COL treatment was more effective than the REHAB treatment, we analyzed the VISA-H variation (delta) and its speed (acceleration; delta VISA-H points per week), using data from the comparison between the two visits (T1–T0), for both treatments. *T*-Tests (parametric and unpaired) were performed for the delta and speed analyses too. The mean delta point variations for the VISA-H results were 18.50 ± 0.4776 and 13.79 ± 1.692 for the COL and REHAB treatments, respectively (Figure 7, right graph), while the mean speed values were 3.085 ± 0.0795 and 2.298 ± 0.388, respectively (Figure 7, Right graph). Both analyses of the difference between COL and REHAB revealed statistically significant differences: delta VISA-H (*p* = 0.0171) and VISA-H speed (*p* = 0.0263). Nonetheless, these differences indicate a positive increase in delta VISA-H scores and VISA-H speed in the COL treatment, with respect to the REHAB treatment (+34.24%), confirming the reduction in delta VISA-H in the clinical evaluation (Figure 7).

### 3.8. Proportion and Hazard Ratio (HR) to RTS

**Figure 8:**
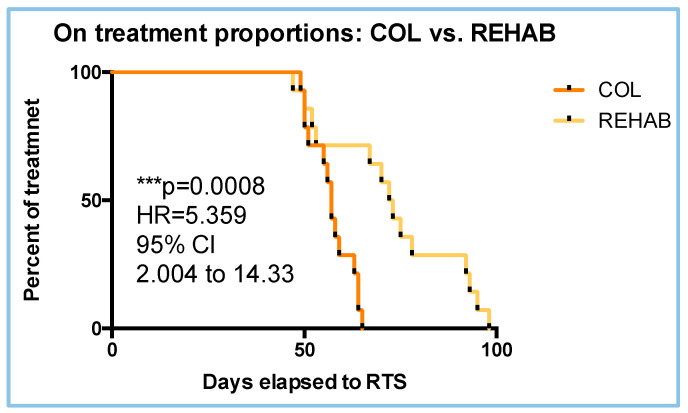
KM proportion for the COL and REHAB populations.

To evaluate whether these two populations differ in terms of proportion of treatment, a Kaplan–Meyer (MK) long-rank analysis was performed. Here, we assumed that a censored event was associated with RTS; indeed, the proportion revealed the percent of athletes still under treatment. The results revealed statistical differences among the population (COL vs. REHAB *p* = 0.0008). Nevertheless, the return to sport (RTS) hazard ratio for the COL treatment was 5.359 (95% CI of ratio: 2.004 to 14.330; Figure 8). These data suggest that the COL treatment increased RTS probability by five times more than the REHAB protocol (Figure 8).

## 4. Discussion

Hamstring injuries (HIs) are among the most common types of injuries affecting athletes and sportive people [5,7,29,54]. Despite optimal management, including diagnosis [23,29] and treatments [5,53,55], the cause of hamstring muscle injuries is not well defined [29,39]. Indeed, differential diagnoses of HSIs are often needed [7]. The principal pathologies associated with the diagnosis of HSIs include proximal hamstring tendinopathy (PHT) [56,57], proximal hamstring tendon avulsion (PHTA) [4], lumbar spine radiculopathy (LSR) [7], and adductor muscle injury (AMI). The manifestation of symptoms includes pain in the back of the thighs, sometimes accompanied by an audible or sensory popping sound, causing immediate cessation of activity [9]. The main symptom of proximal hamstring tendinopathy (PHT) is lower gluteal pain, especially during running or prolonged sitting [57]. According to classification based on HI consensuses [16], 28 athletes affect by PHT were included in this study. The subjects were split into two groups associated with two different treatments: one associated with porcine collagen treatment (COL) and one with a usual rehabilitation program (REHAB) [39]. These traditional treatment methods for PHT are mostly comparable with those of other tendinopathies: they include rest and ice for symptom relief in the initial phase, reduction or pause of sports activity, non-steroidal anti-inflammatory drugs (NSAIDs), soft tissue mobilization, physiotherapy, and continuous home exercise programs focusing on progressive eccentric hamstring strengthening and core stabilization [58]. Nonetheless, surgical approaches are contemplated when conservative procedures fail [24,25,59]. Notably, in such cases, RTS time is prolonged by up to 12 months [24]. Indeed, conservative approaches remain the first choice, if possible [27]. Novel therapeutic approaches have recently been recognized, including shockwave (SWT) [28], ultrasound (US) therapy [30], and ultrasound-guided corticosteroid [31] and platelet-rich-plasma (PRP) injections [33]. The efficacy of collagen application in tendons was first proposed by Randelli et al., who reported the efficacy of porcine collagen in human tenocytes [42]; indeed, other clinical studies have since reported porcine collagen efficacy in humans [36,37,49,60].

The first aim of all abovementioned treatments is to increase benefits for athletes and to reduce their time to return to sport (RTS). Faster treatment times seem to be achieved with corticosteroid injections (RTS = 1 month). However, several clinicians reported several side effects associated with the treatment, including local irritation, skin depigmentation, suppression of tenocyte activity and collagen synthesis, and tendon avulsion [29,61,62]. The other options offer RTS times of 3 (SWT) or 4.5 (PRP) months. In particular, the efficacy–benefit ratio for PRP injections is quite controversial [29,63]. Porcine collagen injections (COL treatment) require the same specialization needed for corticosteroid and plasma injections but demonstrate better results in terms of RTS time. In fact, the mean RTS time of the COL group was 57 days, less than two months. The analyses in this study also included clinical evaluations of the athletes using the VISA-H questionnaire. This procedure is very well documented and is currently used in Europe for football players’ PHT evaluations [57,64]. A normal VISA-H score was evaluated to be around 60.9 (mean value) in this study [64]. The evaluation of VISA-H was positive in both groups after treatment (COL at T1 and REHAB at T1). However, the VISA-H scores for the COL group were better than those for the REHAB one, with its mean score equal to 62.00. These data are in agreement with those present in the literature.

Nonetheless, the COL treatment demonstrated better results (vs. the REHAB group) in terms of RTS time (57 vs. 72 days), delta VISA-H (18.50 vs. 13.79 points), and VISA-H speed (3.085 vs. 2.298 points/week). Finally, the log-rank test revealed a strong *p*-value (*p* = 0.0008) for the ratio of athletes remaining in treatment. Therefore, the analyses revealed an HR = 5.359 for the COL group with respect to the REHAB treatment in reducing RTS time.

Explanations for why collagen has such a positive effect can probably be found in the biological mechanisms involved in the repair process after a hamstring strain injury [39]. A significant increase in the production of type III collagen at the beginning of the healing process has been documented [65]. This type of collagen fibril has a much smaller diameter and, therefore, a lower resistance to tensile force than type I collagen fibrils. This fact implies lower load-bearing capacities compared with healthy muscle tissue, where the percentage of type I collagen is much more abundant [66,67]. As time passes, the ratio of type I/III collagen fibers, i.e., the tensile strength of the repaired tissue, increases significantly [33,65,68]. To provide mechanical support and to form a fiber capable of withstanding physical stress, collagen fibers must be highly cross-linked [65]. Notably, corticosteroid treatments seem to reduce the potential healing around tenocytes [62]. These analyses demonstrate that the two analyzed populations are very homogeneous, and the distribution is parametric. Indeed, the statistical differences demonstrated above seem not to be affected by anagraphical data or the levels of their sport activities. In the context of injection-based therapies for PHT, platelet-rich plasma (PRP) has been the most widely investigated, with evidence suggesting potential benefits through the delivery of growth factors that stimulate tissue regeneration. Several randomized controlled trials and meta-analyses have evaluated PRP in tendinopathy, though the findings remain mixed, with methodological heterogeneity and variable follow-up timing [20,27,69]. Collagen injections may offer complementary or alternative benefits by directly supplying structural proteins to the extracellular matrix (ECM), potentially promoting tendon remodeling and type I/III collagen balance restoration [66,69]. However, the precise mechanisms remain speculative and require further mechanistic research. Nevertheless, the COL approach demonstrated better stratification of the results in terms of athletes’ RTS times and VISA-H scores. Nonetheless, some limitations are present in this study, such as the small sample size (n = 14 per group), the absence of a placebo or sham-injection control group, the lack of comparisons with other injection-based therapies such as PRP or corticosteroids, the lack of long-term follow-up beyond six months, and the small heterogeneity in the types of sports represented. Notably, the corticosteroids infiltration can be dangerous for the tendons and (overall) may be considered a doping action. Furthermore, the absence of side effects and the safety of the COL treatment constitute pathways for the authors to continue investigating to bridge a big part of these limitations. As a general concept, PHT injuries also represent an important economic turning point in the professional discipline where the athlete works. Enormous budget losses are caused by injuries in the world of professional sports (i.e., football). Therefore, RTS time becomes a requirement for sports health, the recovery of athletic performance, reduction in economic losses in terms of medical expenses, and profits.

## 5. Conclusions

Proximal hamstring tendinopathy (PHT) is one of the most important pathologies affecting professional athletes and their performance, causing them to stop their sports activities. The time to return to sport (RTS) is a crucial elapsed timeframe influencing the performance of an athlete and the budget of their staff. Indeed, shorter RTS times change many aspects of professional sports. Conservative approaches (starting from rehabilitation to injective therapies) aim to modify RTS times. Our analyses of new conservative treatments (COL; MD-MUSCLE) seem to demonstrate several advantages with respect to rehabilitation protocol, including (1) a reduction in RTS time, (2) improvements to clinical conditions, and (3) accelerated clinical benefits and more uniform results after treatment. Nevertheless, some limitations are still present in these analyses, such as the low number of athletes in this study and the absence of a placebo group. The porcine collagen injections resulted in a shorter RTS time due to the regenerative and reparative mechanism of action, where the turnover between type I and type III collagen fibers might explain the particular efficacy of collagen injection. However, a larger cohort of athletes needs to be investigated to confirm the clinical benefit of conservative porcine collagen treatment in PHT.

## Figures and Tables

**Table 1:** Shapiro–Wilk test results regarding Figure 2.

Treatment	W-Value	*p*-Value	Parametric Data
COL	0.9573	0.6787	YES
REHAB	0.9583	0.6952	YES

Note. The W- and *p*-values refer to the Shapiro–Wilk normality test.

**Table 2:** Shapiro–Wilk test results regarding Figure 4.

Treatment	W-Value	*p*-Value	Parametric Data
COL	0.9168	0.1974	YES
REHAB	0.9201	0.2268	YES

Note. The W- and *p*-values refer to the Shapiro–Wilk normality test.

**Table 3:** Shapiro–Wilk test results regarding Figure 5.

Treatment	W-Value	*p*-Value	Parametric Data
COL (T0)	0.9573	0.6787	YES
COL (T1)	0.9468	0.5123	YES

Note. The W- and *p*-values refer to the Shapiro–Wilk normality test.

**Table 4:** Shapiro–Wilk test results regarding Figure 6.

Treatment	W-Value	*p*-Value	Parametric Data
REHAB (T0)	0.9583	0.6952	YES
REHAB (T1)	0.9371	0.3820	YES

Note. The W- and *p*-values refer to the Shapiro–Wilk normality test.

## Data Availability

All data obtained from this study are available for consultation. The data controller is Matteo Baldassarri.
